# Physicochemical, Sensory and Digestive Properties of Eel Burgers at Different Baking Temperatures

**DOI:** 10.3389/fnut.2022.923433

**Published:** 2022-06-24

**Authors:** Jieyu Li, Linfan Shi, Zhongyang Ren, Wuyin Weng

**Affiliations:** ^1^College of Ocean Food and Biological Engineering, Jimei University, Xiamen, China; ^2^Engineering Research Center of the Modern Technology for Eel Industry, Ministry of Education, Xiamen, China; ^3^Research Center of Marine Functional Food, Xiamen, China; ^4^Collaborative Innovation Center of Provincial and Ministerial Co-construction for Marine Food Deep Processing, Xiamen, China

**Keywords:** American eel, gel strength, microstructure, flavor, taste, *in vitro* digestion

## Abstract

The effect of baking temperature on the physicochemical, sensory and digestive properties of eel burgers was investigated. The moisture content of eel burgers gradually decreased with increased baking temperature, whereas the water-holding capacity remained unchanged. The breaking force of eel burgers baked at 160°C was significantly higher than that at other baking temperatures. With increased baking temperature from 100 to 220°C, amide I in the Fourier transform infrared spectroscopy of eel burgers shifted from 1,645 to 1,633 cm^−1^, and the peak intensity of 1,744 cm^−1^ initially increased and then decreased. When the baking temperature exceeded 160°C, the band intensity of protein aggregate increased gradually with increased baking temperature. Scanning electron microscopy result indicated that the muscle fibers in eel burgers contracted significantly with increased baking temperature, and a honeycomb-like network structure appeared in eel burgers baked at 220°C. The sulfur compounds in the eel burgers baked at 130°C were lower than those of the sample baked at 100°C, but it increased gradually with further increased baking temperature. The aftertaste astringency, richness, saltiness, and overall acceptability of eel burgers increased with increased baking temperature. The eel burgers baked at 130–160°C could be easily digested according to the *in vitro* digestibility and confocal laser confocal microscopy of gastrointestinal digests. In conclusion, the texture properties, barbecue aroma, and digestibility of eel burgers could be controlled by the baking temperature.

## Introduction

Eels are a typical warmwater fish with 19 species, among which Japanese (*Anguilla japonica*), American (*Anguilla rostrata*), European (*Anguilla anguilla*), and Australian (*Anguilla australis*) eels are commercially important (1). The annual eel production in China has exceeded 250,000 t since 2020, and American eels have the highest proportion among the different regions due to the comprehensive consideration of product quality and cost. Eels are usually roasted like *kabayaki* according to the market demand and high-fat characterization. Heating is a crucial part of commercial food processing as it highly affects the nutritional value, flavor, and texture of products (2).

The mechanisms involved in protein gelation are often determined to investigate the relationship between the gel texture and functional properties of proteins (3). During superheated steam cooking, muscle-fiber integrity is gradually destroyed by increased hot-air cooking temperature, and the structure of muscle-fiber bundle even almost disappears at 180°C (4). At high temperatures, the cross-linking of proteins occurs after being roasted, and the roasting-induced protein aggregation increases with increased temperature (5). The number of protein carbonyls in chicken patties roasted at 170°C is reportedly lower than that of samples boiled in a hot water bath due to that the Maillard reaction products formed at temperatures above 100°C inhibit the protein oxidation (6). On the other hand, the addition of lipid could improve the juiciness, tenderness, and mouthfeel of composite gel products (7). Volatile compounds generated by the Strecker degradation of amino acids and oxidation of lipids could provide roast odor and delicious flavors (8). After being baked, the aromatic profile of farmed sea bass containing a high lipid content is much richer than that of wild sea bass (9). The mushroom-like odor provided by 1-octen-3-ol is found in a red mullet product oven cooked at 200°C (10). The content of alcohols, aromatic compounds, and sulfur compounds in grilled eels is reportedly significantly higher than that of fresh eels (11). A previous study has suggested that the baking temperature plays an important role in the formation of flavor compounds, and the roasted aroma could be increased by increasing temperature (12). However, the effect of baking temperature on the physicochemical, sensory and nutrition properties of eel burgers is rarely reported.

Electronic-nose and electronic-tongue systems, which can convert the flavor information of products into numerical values, are extensively used in the food-flavor evaluation (13). In the present study, the effect of baking temperature on the physicochemical properties of eel burgers from American eels (*Anguilla rostrata*) was investigated, and the flavor characteristics of baked eel burgers were evaluated by using an electronic nose and electronic tongue combined with sensory analysis.

## Materials and Methods

### Materials and Chemicals

Pepsin from porcine gastric mucosa (250 U/mg) and α-chymotrypsin from bovine pancreas (1,000 U/mg) were purchased from Sigma-Aldrich Co. (St. Louis, USA). Trypsin (50,000 U/g) was purchased from Sinopharm Chemical Reagent Ltd., Co. (Shanghai, China). Lipase from Candida rugosa (700 U/mg) was purchased from Beijing Solarbio Sciences & Technology Co., Ltd. (Beijing, China). Bile salt from pig was purchased from Shanghai Macklin Biochemical Co., Ltd. (Shanghai, China). Live eels (*Anguilla rostrata*) with an average weight of 1 kg were purchased from a local supermarket. Upon arrival, eels were placed on ice and trimmed to remove head, skin, bones, and viscera. The obtained white meat was collected to mince with 4% (w/w) sorbitol, 4% (w/w) sucrose, 0.2% (w/w) sodium tripolyphosphate, and 0.2% (w/w) sodium pyrophosphate. The prepared paste was stored at −20°C for further use. Sorbitol, sucrose, NaCl, sodium tripolyphosphate, and sodium pyrophosphate were food-grade, and all the other chemical agents were of analytical grade.

### Preparation of Eel Burgers

To prepare eel burgers, eel paste was firstly thawed at 4°C overnight and ground in a mortar by using a pestle for 5 min, which was followed by an extra 15 min of grinding after adding 2% (w/w) NaCl. The mixture was then molded overnight at −20°C and baked at 100, 130, 160, 190, and 220°C for 30 min using an oven (SK-3O30, Sanki Co., Japan), respectively. After baking, the eel burgers were cooled with ice and stored at 4°C until analyzed.

### Moisture Content and Water Holding Capacity

Two grams of eel burger were precisely weighed, and moisture content was gravimetrically determined by drying to constant weight at 105°C. Water holding capacity (WHC) was determined using the method of Fang et al. (14). About two grams of eel burgers were wrapped with filter paper, placed in centrifuge tubes, and centrifuged at 4°C and 1,000 g for 15 min. WHC was determined as the mass ratio of eel burgers before and after centrifugation.

### Textural Properties

Textural properties were tested using a texture analyzer (Stable Micro System, TA-XT Plus, UK) equipped with a spherical plunger (diameter 5 mm) and 50 N load cell according to a previous method (14). The test speed, pre-test speed, post-test speed, trigger force and distance were 0.5 mm/s, 0.5 mm/s, 1.0 mm/s, 5 g and 10 mm, respectively. The breaking force (g) and defomation (mm) of eel burgers were obtained at room temperature.

### SDS-PAGE

The samples of eel burgers were dissolved in a protein-denatured solution (8 mol/L urea, 2% SDS, and 20 mmol/L Tris-HCl; pH 8.8) with and without 2% β-mercaptoethanol (β-ME) (15). After the solution was centrifuged at 10,000 g and 25°C for 15 min, the obtained supernatant was mixed with SDS-PAGE sample buffer containing 4% SDS, 20% glycerol, with or without 10% β-mercaptoethanol, and 0.125 mol/L Tris-HCL (pH6.8). Electrophoresis was performed using 4% stacking gel and 6% seperating gel.

### FT-IR Analysis

The FT-IR spectra of eel burgers were obtained using an FT-IR spectrometer (Nicolet, iS50, MA, USA). The eel burgers were placed on an attenuated total reflectance (ATR) crystal after being lyophilized (16). The scanning wavelengths ranged from 4,000 to 1,000 cm^−1^ with a resolution of 4 cm^−1^.

### Scanning Electron Microscopy (SEM)

The microstructures of samples were observed following the method of Weng and Zheng (15). The eel burgers were cut into 2 × 2 × 2 mm^3^ pieces, immersed in 0.1 mol/L phosphate buffer (pH 7.2) containing 2.5% glutaraldehyde (v/v) and immobilized in darkness for 24 h. It was then washed with 0.1 mol/L phosphate buffer (pH 7.2), followed by gradient dehydration with 30, 50, 60, 70, 80, 90, and 100% ethanol. After dehydrating, the samples were dried at the critical point using CO_2_ as a transition fluid. The prepared samples were mounted onto aluminum specimen holders coated with gold and photographed with an SEM system (Hitachi, S-4800, Tokyo, Japan).

### Electronic-Nose Analysis

The flavor characteristics of eel burgers were analyzed using an electronic nose (Airsense, PEN 3.5, Schwerin, Germany) according to the method of Yin et al. (17). The electric-nose system comprised 10 metal-oxide gas sensors including W1C (aromatic compounds), W1S (methyl), W1W (sulfides), W2S (alcohols, aldehydes and ketones), W2W (organic sulfides, aromatic compounds), W3C (ammonia, aromatic compounds), W3S (long-chain alkanes), W5C (short-chain alkanes, aromatic compounds), W5S (nitrogen oxides), and W6S (hydrides). For the analysis, 4 g of minced eel burger was placed in 20 mL airtight vials and then incubated in a water bath at 50°C for 60 min. Headspace gas was pumped through the sensor array at 400 mL/min to change the sensor conductance.

### Electronic-Tongue Analysis

Taste attributes were analyzed using an electronic tongue (Insent, TS-5000Z, Atsugi-shi, Japan) according to the method of Yin et al. (17) and Yang et al. (18) with minor modifications. The bitterness, astringency, aftertaste, richness, and saltiness of each sample were measured at 25°C. For the analysis, minced eel burgers (60 g) were mixed with four volumes (w/v) of distilled water (240 mL) and then homogenized at 13,000 rpm for 1 min by using a homogenizer (Fluko, FA25, Shanghai, China). The mixture was incubated in a water bath at 50°C for 60 min and centrifuged at 10,000 g for 10 min at 4°C. The supernatant was passed through filter paper twice for subsequent analysis.

### Sensory Evaluation

A panel of 10 members with training experience was asked to assess the organoleptic properties of the cooked eel burgers according to the method of Al-Juhaimi et al. (19) with minor modifications. Scores were determined using a 9-point scale as follows. Color was rated 1 for white or motley with black spots, 5 for uneven light yellow with black spots, and 9 for uniform golden yellow without black spots. Flavor was rated 1 for heavy fishy or peculiar odor, 5 for the light aroma of grilled eel with peculiar odor, and 9 for the strong aroma of grilled eel without peculiar odor. Taste was rated 1 for loose texture without elasticity, too hard or too soft, greasy; 5 for less firm texture or elastic, less hard or less soft, a little greasy; and 9 for firm, elastic, moderate hardness, not greasy. Juiciness was rated 1 for too juicy or too dry, 5 for a little juicy, and 9 for acceptable juicy. Overall acceptability was rated 1 for dislike extremely, 5 for a little like, and 9 for like extremely.

### *In vitro* Digestion

The *in vitro* digestion model was established according to the methods of Kim et al. (20) with minor modifications. The baked eel burgers (50 g) were ground with 50 ml of distilled water. The obtained mixture was added with 100 ml gastric juice (6.9 mmol/L KCl, 0.9 mmol/L KH_2_PO_4_, 72.2 mmol/L NaCl, 0.12 mmol/L MgCl_2_, 0.5 mmol/L (NH_4_)_2_CO_3_, 0.15 mmol/L CaCl_2_, 4,000 U/mL pepsin), then adjusted to pH 3.0 with HCl, and stirred at 37°C for 2 h. The obtained gastric digests were added with 200 ml of intestinal juice (6.8 mmol/L KCl, 0.8 mmol/L KH_2_PO_4_, 123.4 mmol/L NaCl, 0.33 mmol/L MgCl_2_, 0.6 mmol/L CaCl_2_, 200 U/mL trypsin, 50 U/mL α-chymotrypsin, 2,000 U/mL lipase, 8.6 g/L bile salt), then adjusted to pH 7.0 with NaOH, and stirred at 37°C for 2 h. After digestion, the gastrointestinal (GI) digests were heated at 90°C for 15 min and cooled with ice. The supernatant was collected by centrifugation (10,000 g, 15 min, 25°C) and freeze-dried. The control was conducted in the same manner without centrifugation by adding the gastric juice and intestinal juice which were heated at 90°C for 15 min before addition. The digestibility of baked eel burgers was calculated using the following equation:


(1) $$\begin{array}{r} Digestibility\;\left(\%\right)=\left[\frac{W_{s}-\left(W_{c}-W_{e}\right)}{W_{e}}\right]\times 100 \end{array}$$

Where *W*_*s*_, *W*_*c*_ and *W*_*e*_ refer to the weight of freeze-dried supernatant from GI digests, freeze-dried control samples and baked eel burgers before digestion, respectively.

The morphology of GI digests was observed using a Leica TCS SP8 confocal laser confocal microscopy (CLSM; Leica, Germany) according to a method reported by Cao et al. (21). The GI digests (1 mL) were mixed with 5 μL of mixed staining solution containing Nile red (0.05%, w/v) and FITC (0.05%, w/v). The obtained mixture was observed using the 40 × objective. The excitation wavelengths were 488 and 543 nm for Nile red (mark of fat) and FITC (mark of protein), respectively.

### Statistical Analysis

All data are presented as the mean ± standard deviation. ANOVA was used to analyze the data. Differences among the levels were measured by Duncan's multiple-range test (*P* < 0.05) with SPSS software (version 17.0, SPSS, Inc., Chicago, IL, USA).

## Results and Discussion

### Moisture Content and WHC

**Figure 1:**
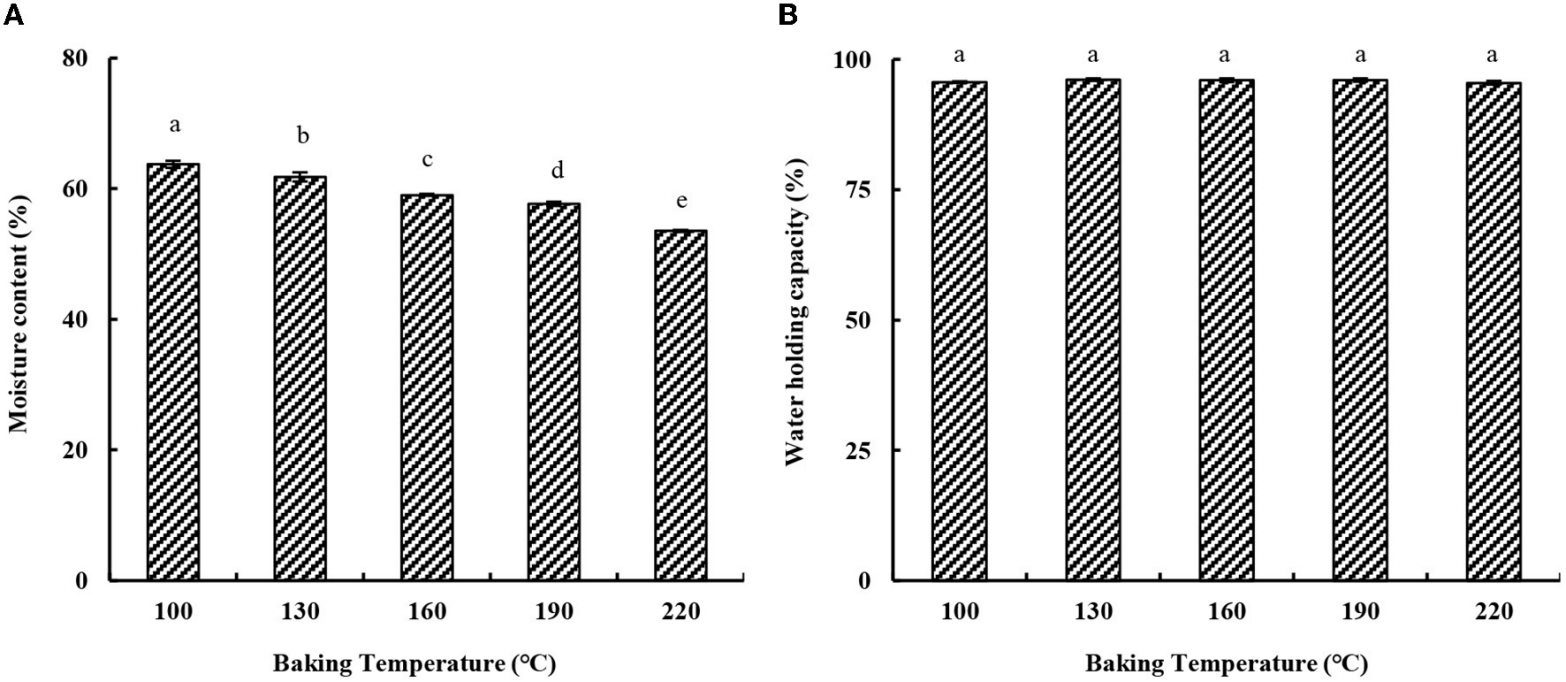
Effect of baking temperature on the moisture content **(A)** and water holding capacity **(B)** of eel burgers.

The moisture content of eel burgers is shown in Figure 1A. The moisture content of eel burgers baked at 100°C was 63.75%, which was slightly lower than that of paste (66.56%) due to water evaporation during baking. The moisture content of eel burgers gradually decreased with increased baking temperature. A previous study has shown that the moisture content of European eels decreased from 47.4 to 32.0% after baking at 200°C for 15 min (22). In this study, the moisture content of eel burgers decreased by about 10% after being baked at 220°C for 30 min probably due to the improved water-maintaining ability of the added sugar and phosphate in eel paste.

WHC is a key indicator of surimi gel quality. As shown in Figure 1B, the WHC of eel burgers was about 95.6% irrespective of the baking temperature of 100–220°C. Regarding the effects of different concentrations of Camellia tea oil on surimi gel physicochemical properties, oil occupies the void spaces of the protein matrix and forms a firmer structure that could trap more water (23). No obvious effect of baking temperature on the WHC of eel burgers was found in the present study probably due to the high-fat content (Figure 1B), which may have formed an external hard layer on the surface of baking sample to inhibite the exit of seeping liquid (24). It was also reported that baking yielded a tilapia fish burger with high WHC (95.82 ± 0.77), which was not affected by the baking and grilling methods (25).

### Textual Properties

The breaking force and deformation of eel burgers baked at different temperatures are presented in Table 1. The breaking force of eel burgers baked at 100°C was 67.76 g, which significantly increased with increased baking temperature and reached the highest value at 160°C. This finding was due to the unfolding of protein promoted by an appropriate increase in baking temperature, thereby improving the interaction among heat-denatured proteins (2). Based on the changes of sulfhydryl group content, a previous study suggested that protein aggregation in the hairtail filets could be formed through new disulfide bonds and hydrophobic interactions during baking at 220°C (26). However, with further increased baking temperature, the breaking force of eel burgers showed a downward trend (Table 1). Notably, the fat of sardine filets baked at 200°C decreased from 15.44 to 14.60% compared with raw samples (27). Therefore, the fat could be released from eel muscle at high-temperature baking and filled into the protein-network structure, causing the breaking force to decrease with increased deformation of eel burgers.

**Table 1:** Effect of baking temperature on the gel strength of eel burgers.

**Baking temperature (**°**C)**	**Breaking force (g)**	**Deformation (mm)**
100	67.76 ± 27.65^d^	6.20 ± 1.15^d^
130	287.29 ± 34.86^bc^	8.30 ± 0.61^c^
160	400.07 ± 33.04^a^	9.19 ± 0.23^bc^
190	327.84 ± 31.82^b^	9.58 ± 0.20^b^
220	256.59 ± 41.87^c^	9.90 ± 0.22^a^

*Data are expressed as mean ± standard deviation. Values with different letters in the same column indicate significant differences at P < 0.05*.

### SDS-PAGE

**Figure 2:**
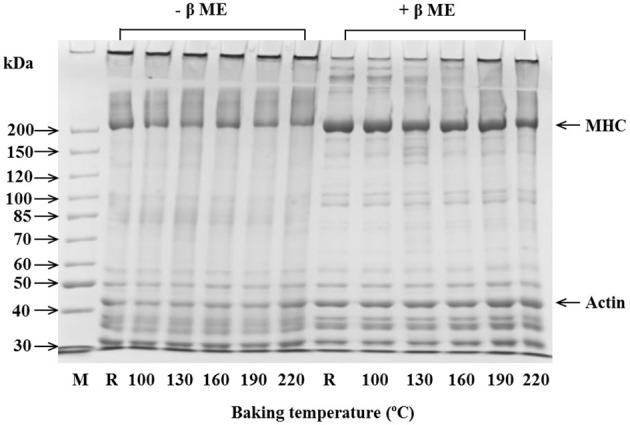
SDS-PAGE of eel patties and burgers at various baking temperatures. M, protein standard; R, eel patties.

The electrophoresis analysis of eel paste and burgers is shown in Figure 2. Myosin heavy chain (MHC, 200 kDa) and actin (43 kDa) were the main components of eel paste. Regardless of baking temperature, no changes occurred in the band intensities of MHC and actin of the eel burgers, and a large amount of high-molecular-weight polymer (HMWF) too large to enter the polyacrylamide gel was observed. Meanwhile, when the electrophoresis samples were added with β-ME, the band intensity of HMWF weakened, whereas the band intensity of MHC and actin slightly increased, indicating that eel paste and baked burgers contained disulfide bonds. When the eel burgers were baked at 160°C or above, the band intensity of HMWF gradually increased, indicating that some HMWF could be formed through non-covalent bonds at excessive baking temperatures. A similar phenomenon has been reported about the effect of different cooking methods on the physicochemical properties of farmed sturgeon filets (28).

### FT-IR Analysis

**Figure 3:**
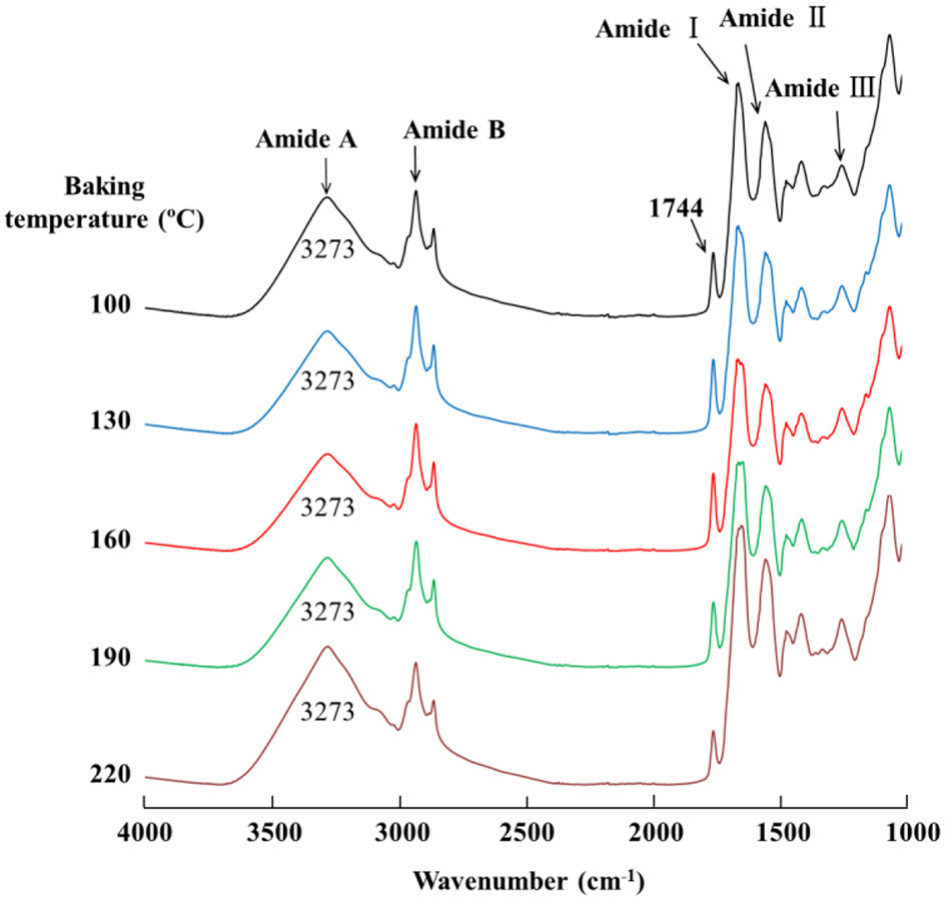
FTIR spectra of eel burgers baked at various temperatures.

The FT-IR spectra of eel burgers are shown in Figure 3. No obvious changes occurred in the absorption peaks of amide A, B, II, and III among the eel burgers baked at various temperatures. Notably, the wavenumber 1,744 cm^−1^ did not change, but its peak intensity increased and then decreased with increased baking temperature from 100 to 220°C. Conversely, the amide I band of eel burgers baked at 100–190°C was found at 1,645 cm^−1^, which shifted to 1,633 cm^−1^ when eel burgers were baked at 220°C.

Amide A (3,000–3,600 cm^−1^), attributed to N-H and O-H stretching vibrations, was related to changes in the hydrogen bond (29). Amide B (2,800–3,000 cm^−1^) and the absorption band of 1,740–1,760 cm^−1^, associated with C-H and C=O stretching vibrations, respectively, represented lipid change (30). Amide I (1,600–1,700 cm^−1^) was assigned to C=O stretching vibration, whereas amide II (1,480–1,575 cm^−1^) and amide III (1,229–1,301 cm^−1^) were assigned to N-H bending and C-N stretching vibration, respectively (29). Moreover, the displacements of amide I, II, and III can represent the change in inter/intramolecular hydrogen bonds of protein (31). Based on the results of amides A, B, II, and III shown in Figure 3, no obvious effects of baking temperature on the hydrogen bond of the protein molecules in eel burgers were found. The changes in the peak intensity of 1,744 cm^−1^ suggested that the fat content of eel burgers initially increased and then decreased with increased baking temperature. This finding could be due to the gradual release of fat from the eel muscle with increased baking temperature from 100 to 160°C, but fat might lose slightly with further increased baking temperature. A similar trend was observed in chicken sausage superheated steam cooked at various temperatures (150, 200, and 250°C) with different time (32).

### Microstructures

**Figure 4:**
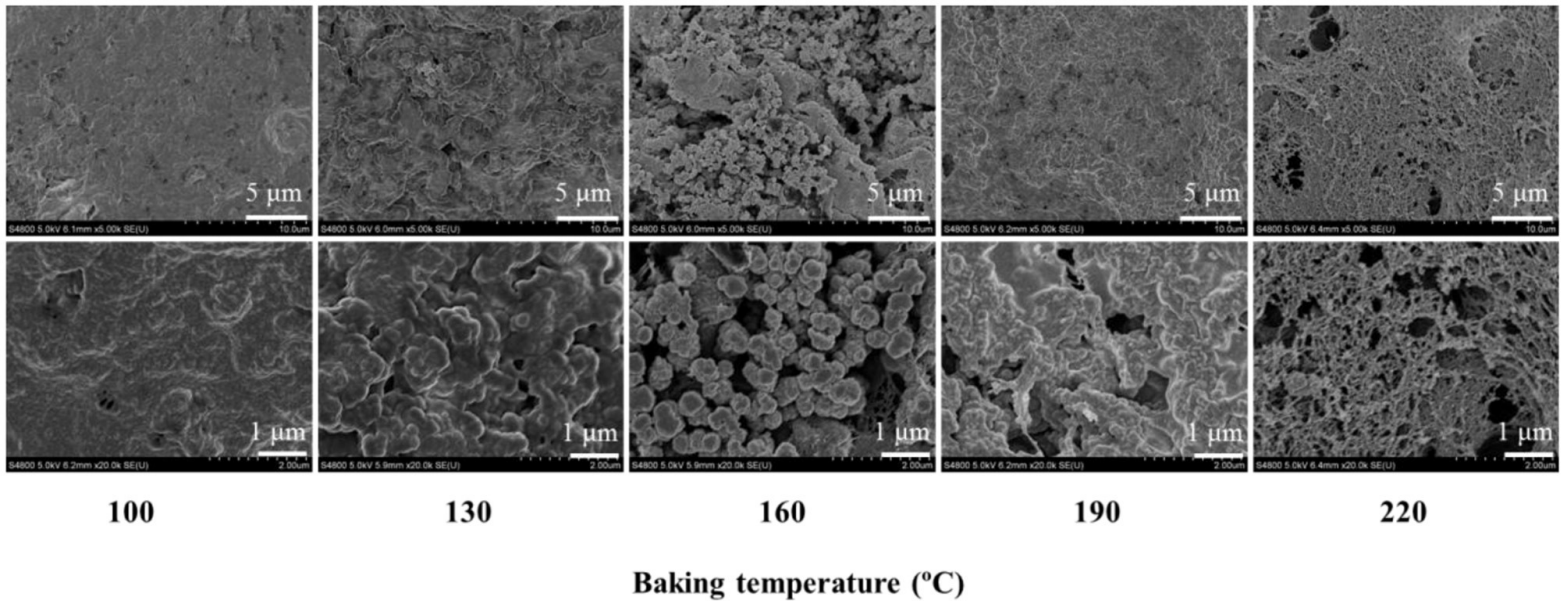
Microstructure of eel burgers baked at various temperatures.

The microstructures of eel burgers baked at various temperatures were characterized by SEM as shown in Figure 4. The fiber tissue in the eel burgers baked at 100°C was arranged neatly and densely, with a clear muscle texture. With increased baking temperature to 130°C, the muscle fibers in the eel burgers shrank to a certain extent, and a layer of oil-like luster appeared on the surface probably due to the part of fat released from muscle tissue. This finding was consistent with a previous report stating that the protein of foal meat is denatured and the fat is gradually released during baking, resulting in tissue-structure reorganization of the roasted product (24). When the eel burgers were baked at 160°C, aggregates and pores of different sizes formed probably due to the excessive shrinkage of fiber tissue. This finding was similar to the study which reported that pork muscle fibers were destroyed by hot-air cooking, causing the contraction and rupture of the muscle bundle (4). Analysis of the eel burgers baked at 190°C (Figure 4) revealed that the pores formed by fiber shrinkage disappeared partially. This result may be due to the filling of some pores with fat dissolved at high baking temperatures. With further increasing baking temperature, a honeycomb-like fiber structure of baked eel burgers was found probably due to the degradation of myofibrils caused by high baking temperature. Consequently, the gel strength and moisture content of eel burgers significantly decreased (Table 1; Figure 1). In a review on the effects of heat treatment on the protein modification of meat products (2), the aromatic residues of the beef protein are degraded significantly at high heating temperature, thereby affecting the texture qualities of meat products. The results of Figure 4 also showed that the degree of muscle fiber contraction and fat loss increased with increased baking temperature, whereas protein degradation and muscle-fiber tissue destruction were observed in the microstructure of eel burgers baked at high temperature.

### Electronic-Nose Analysis

**Figure 5:**
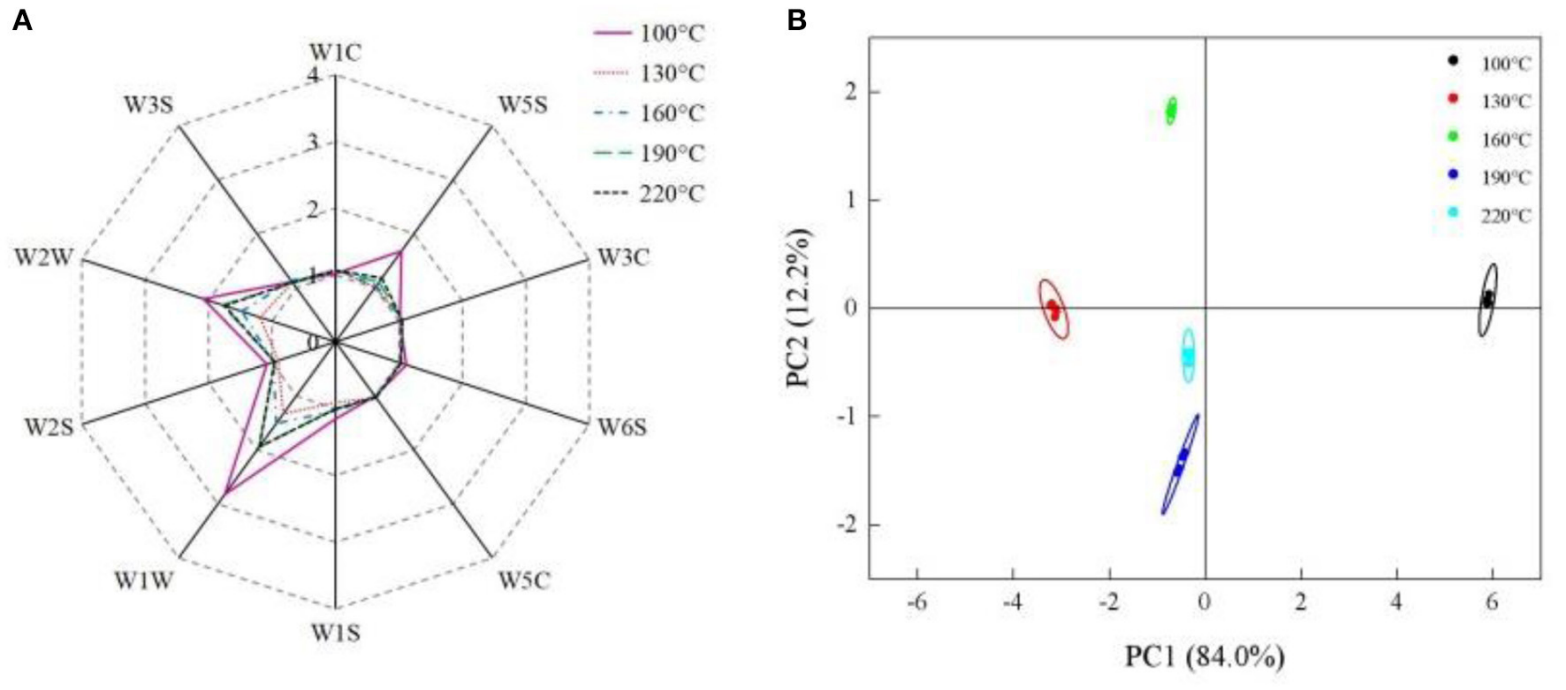
Electronic-nose response data radar chart **(A)** and principal component analysis plot **(B)** of eel burgers baked at various temperatures.

The sensors of the electronic-nose system are sensitive to the aroma and volatile compounds. As shown in Figure 5A, when the eel burgers were baked at 100°C, the response values of W5S, W1W, and W2W were significantly higher than those of other sensors, indicating that nitrogen oxides and sulfides were the main flavor source of the eel burgers baked at 100°C. A similar phenomenon has been reported by Sun et al. who studied the flavor of bigeye tuna meat heated at 100°C for 1 h (33). The response values of W5S, W1W, and W2W of the eel burgers baked at 130°C were significantly lower than the sample baked at 100°C (Figure 5A). As a previous report, the sensors of W5S, W1W, and W2W are reportedly sensitive to aldehydes, with response values reaching a high level in bigeye tuna meat heated at 100°C but decreasing with further increased heating temperature (33). With increasing baking temperature from 130 to 220°C, no change in the response value of W5S was found, whereas the response values of W1W and W2W gradually increased (Figure 5A). W1W and W2W sensors were also sensitive to sulfur compounds, some of which (e.g., dimethyl trisulfide) could provide the characteristic aroma of seafood (11). This finding was consistent with the present results that a higher baking temperature corresponded with a stronger aroma of baked eel burgers.

Figure 5B shows the principal component analysis (PCA) of the electronic-nose sensor data. The first principal component (PC1) and the second principal component (PC2) accounted for 84.0 and 12.2% of the total variance, respectively, indicating that they can be used to analyze the odor information of eel burgers baked at various temperatures (34). The lowest PC1 value was found in eel burgers baked at 130°C, followed by samples baked at 160–220°C, consistent with the trend in Figure 5A. Conversely, eel burgers baked at 160–220°C can be well distinguished on PC2, although the samples baked at 190°C were relatively close to those baked at 220°C. These results showed that eel burgers baked at 160–220°C had similar flavor compounds which could be distinguished by the electronic nose.

### Electronic-Tongue Analysis

An electronic-tongue system was used to detect the taste properties of eel burgers (Table 2). In eel burgers baked at 100°C, the bitterness value was the highest, followed by the astringency value and saltiness value. At 130–190°C baking temperature, no significant changes were found in the bitterness and astringency of eel burgers, which were higher than those of eel burgers baked at 100°C. With increasing baking temperature from 190 to 220°C, the bitterness of eel burgers decreased. Bitter amino acids and bitter peptides are reportedly degraded during thermal treatment at 140°C, leading to decreased bitterness (35). The bitterness of chicken patties baked at 190°C is also higher than that of samples baked at 160°C due to the more intense Maillard reaction and caramelization reaction at 190°C (36). It was reported that bitter amino acids make could make umami taste soft (37). In the present study, the astringency aftertaste-A, richness, and saltiness of eel burgers showed an upward trend with the increase in baking temperature, which may be related to the water evaporation.

**Table 2:** Electronic-tougue analysis of eel burgers baked at various temperatures.

**Baking temperature (**°**C)**	**Bitterness**	**Astringency**	**Aftertaste-B**	**Aftertaste-A**	**Richness**	**Saltiness**
100	6.82 ± 0.05^b^	2.31 ± 0.03^b^	1.20 ± 0.15^a^	1.00 ± 0.02^e^	0.86 ± 0.03^b^	1.96 ± 0.03^c^
130	7.34 ± 0.05^a^	2.62 ± 0.02^a^	1.21 ± 0.45^a^	1.23 ± 0.06^d^	0.79 ± 0.02^c^	1.80 ± 0.01^d^
160	7.32 ± 0.05^a^	2.65 ± 0.03^a^	1.33 ± 0.31^a^	1.28 ± 0.01^c^	0.85 ± 0.02^b^	1.90 ± 0.01^c^
190	7.26 ± 0.01^a^	2.66 ± 0.02^a^	1.42 ± 0.31^a^	1.37 ± 0.02^b^	0.88 ± 0.02^b^	2.12 ± 0.01^b^
220	6.80 ± 0.01^b^	2.66 ± 0.03^a^	1.60 ± 0.36^a^	1.53 ± 0.02^a^	0.95 ± 0.02^a^	2.50 ± 0.02^a^

*Data are expressed as mean ± standard deviation. Values with different letters in the same column indicate significant differences at P < 0.05*.

**Figure 6:**
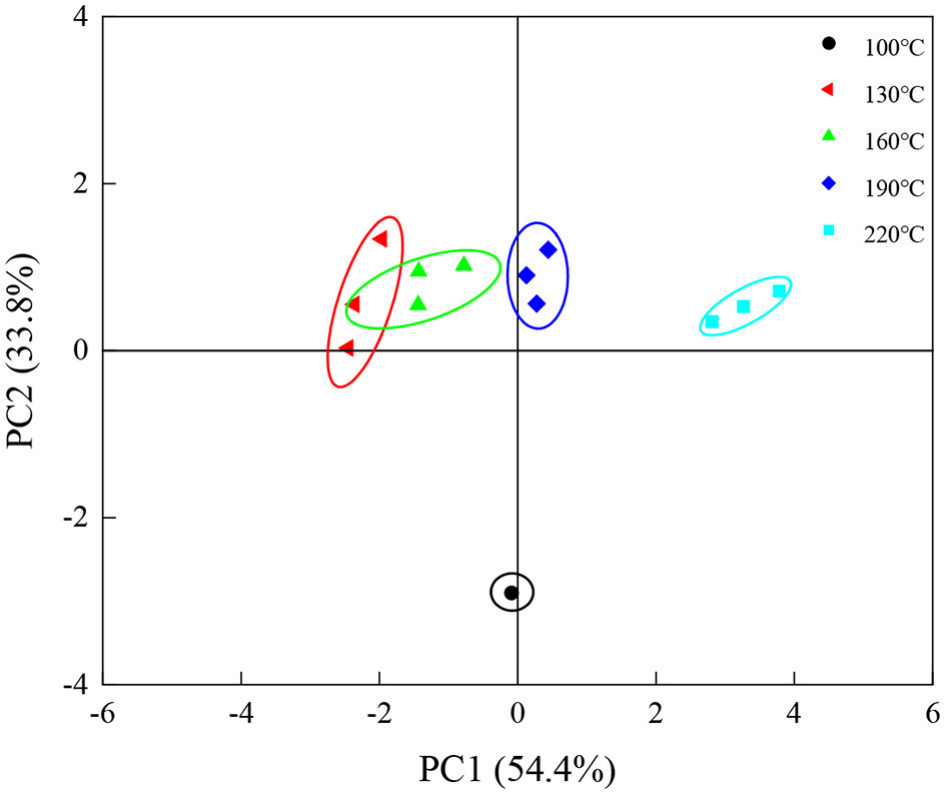
Electronic-tongue principal component analysis plot of eel burgers baked at various temperatures.

Figure 6 shows the PCA results of electronic-tongue data. The total variance contribution rate of PC1 and PC2 was 88.2%, which sufficiently reflected the taste information of baked eel burgers. With increased baking temperature from 130 to 220°C, the PC1 value of eel burgers gradually increased. Among the eel burgers baked at 130–220°C, no significant difference was found in PC2 value, which was clearly distinguished from the eel burgers baked at 100°C. These results indicated that the taste compounds of eel burgers baked at 100°C were different from that of samples baked at 130–220°C, and the taste compounds of the eel burgers baked at 130–220°C could also be distinguished by the electronic tongue.

### Sensory Evaluation

The sensory-evaluation results of eel burgers baked at various temperatures are shown in Table 3. With increased baking temperature, the color score of eel burgers gradually increased. The color change was related to the Maillard reaction primarily due to the condensation of carbonyl and amine to form brown high-molecular-weight compounds (38). The highest flavor score was found in eel burgers baked at 190°C, whereas the highest taste score and juiciness score were found in eel burgers baked at 160°C. These values decreased with increased or decreased baking temperature. Similar trends were observed in the results of electronic nose, electronic tongue, and gel strength (Figure 5, Tables 1, 2). With the increase in baking temperature from 100 to 220°C, the overall acceptability of eel burgers gradually increased, indicating that the color played an important role in the sensory quality of baked eel burgers. With the increase in baking temperature from 100 to 160°C, the fishy odor of eel burgers decreased, whereas the barbecue aroma increased. Meanwhile, the interaction between lipid and protein was promoted by baking. Thus, the increase in baking temperature positively affected the taste and flavor of eel burgers. With further increased baking temperature, the produced burnt flavor negatively affected the flavor and juiciness of eel burgers.

**Table 3:** Sensory evaluation of eel burgers baked at various temperatures.

**Baking temperature (**°**C)**	**Color**	**Flavor**	**Taste**	**Juiciness**	**Overall acceptability**
100	2.80 ± 0.92^c^	3.00 ± 0.82^c^	1.70 ± 1.06^c^	1.70 ± 0.67^c^	1.90 ± 1.10^c^
130	3.20 ± 1.23^c^	4.10 ± 0.88^c^	3.90 ± 1.60^b^	3.30 ± 1.16^b^	2.50 ± 0.97^c^
160	4.70 ± 1.42^b^	6.60 ± 1.35^b^	8.10 ± 0.88^a^	7.80 ± 1.23^a^	4.95 ± 2.11^b^
190	8.40 ± 0.70^a^	8.25 ± 0.92^a^	7.60 ± 0.97^a^	4.00 ± 2.62^b^	8.40 ± 0.52^a^
220	8.60 ± 0.52^a^	3.60 ± 2.22^c^	7.20 ± 0.63^a^	2.70 ± 0.82^bc^	7.40 ± 1.07^a^

*Data are expressed as mean ± standard deviation. Values with different letters in the same column indicate significant differences at P < 0.05*.

### *In vitro* Digestibility

**Figure 7:**
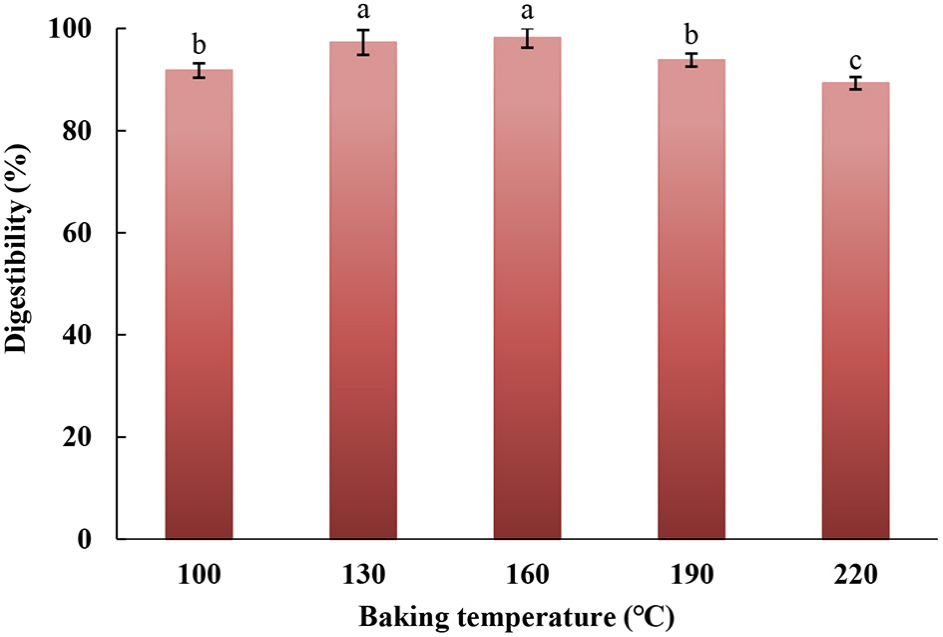
*In vitro* digestibility of eel burgers baked at various temperatures.

The *in vitro* digestibility of eel burgers baked at various temperatures is shown in Figure 7. Among the baked eel burgers, the digestibility of samples baked at 160°C was the highest, reaching up to 98.17%. A hard dry layer could be formed on the surface of the eel burgers with increasing baking temperatures, resulting in the reduction of digestibility. However, the digestibility of the eel burgers baked at 220°C could reach up to 89.3%, which was close to the digestibility (89%) of hairtail filets baked at 220°C for 25 min reported by Semedo Tavares et al. (26).

**Figure 8:**
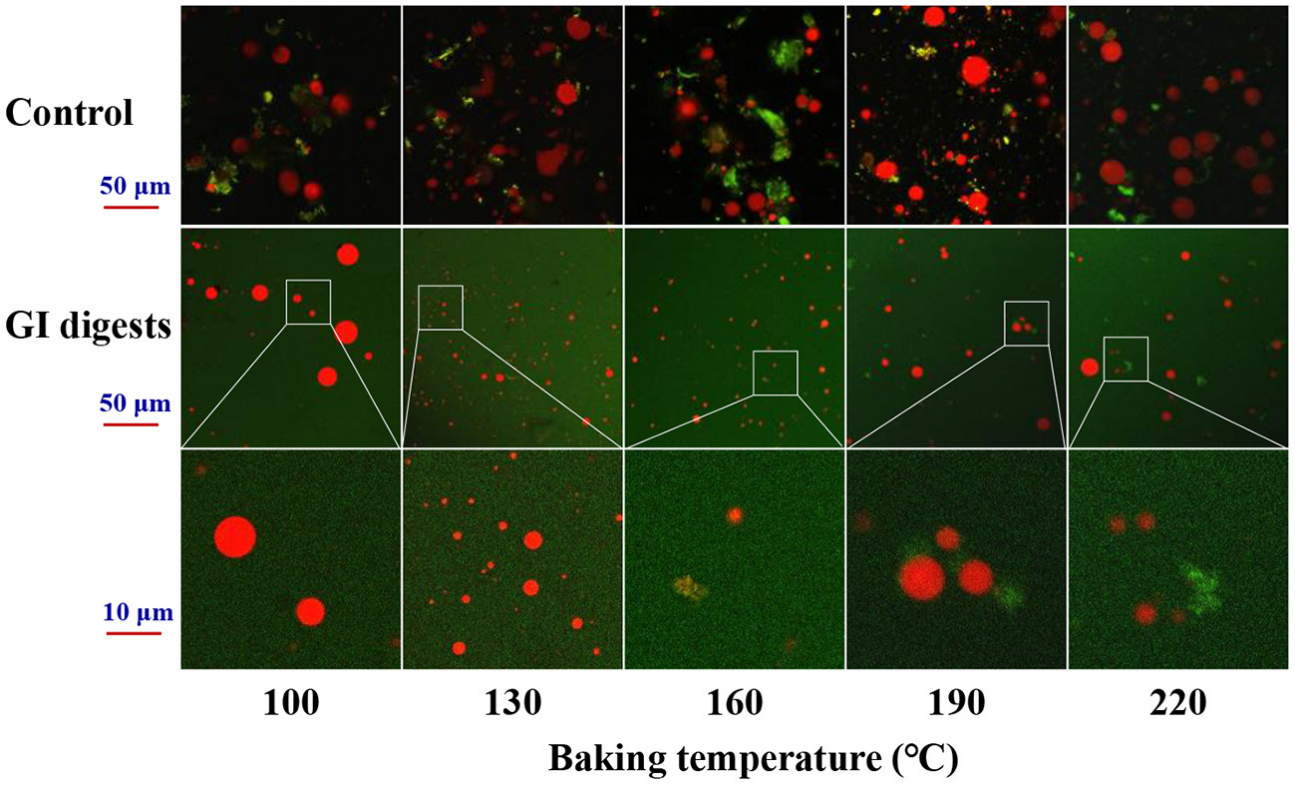
CLSM images of GI digests from baked eel burgers *in vitro* digestion.

The CLSM images of *in vitro* GI digests from baked eel burgers are presented in Figure 8. The red and green fluorescent signals represented fat and protein, respectively. In the control samples, it could be found that fat particles were dispersed in irregular protein fiber fragments. As the baking temperature increased, the fat gradually migrated from the inside of the eel burgers to the outside, showing that the red fluorescence signals increased. After simulated digestion, the dispersed fat particles around the protein phase were found in the GI digests from eel burgers baked at 100°C and the size of some fat particles was slightly smaller than the control. Moreover, the size of fat particles in the GI digests from eel burgers baked at 130 and 160°C was significantly smaller than that of other samples. A review reported that the smaller the fat globule size, the easier it is to be digested (39). When the baking temperature was higher than 160°C, there were little green fluorescence fragments representing protein aggregates in Figure 8 (indicated by magnification), suggesting that indigestible products could be formed during high-temperature baking. These observations were consistent with the finding on the digestibility of baked eel burgers (Figure 7), further suggesting that the proteins and oil of eel burgers baked at 130–160°C could be easily digested and absorbed.

## Conclusion

Baking temperature significantly affected the quality of eel burgers. The increased baking temperature could promote the protein interaction of eel burgers, resulting that breaking force was increased from 67.76 to 400.07 g at 100–160°C. However, when the baking temperature exceeded 160°C, fat was easily released from the muscle tissue and filled into the protein-network structure, and the muscle fibers shrank and broke, resulting in decreased gel strength. Electronic nose and electronic tongue could distinguish the flavors of eel burgers baked at various temperatures. The increase in baking temperature could reduce the fishy odor of eel burgers but enhanced barbecue flavor, astringency, richness, and saltiness. The eel burgers baked at 160°C were conducive to the digestion and absorption of protein and oil, based on 98.17% of *in vitro* digestibility and the smallest sizes of GI digest particles. Therefore, the texture, flavor, and digestibility of eel burgers can be controlled by the baking temperature, thereby affecting the consumers' acceptance of products.

## Data Availability Statement

The original contributions presented in the study are included in the article/supplementary material, further inquiries can be directed to the corresponding author/s.

## Author Contributions

All authors listed have made a substantial, direct, and intellectual contribution to the work and approved it for publication.

## Funding

This work was sponsored by Fujian Key Project of Natural Science Fund (2019J02013) and Fujian Project of Marine Economy Development (FJHJF-L-2021-3).

## Conflict of Interest

The authors declare that the research was conducted in the absence of any commercial or financial relationships that could be construed as a potential conflict of interest.

## Publisher's Note

All claims expressed in this article are solely those of the authors and do not necessarily represent those of their affiliated organizations, or those of the publisher, the editors and the reviewers. Any product that may be evaluated in this article, or claim that may be made by its manufacturer, is not guaranteed or endorsed by the publisher.
